# Mucus increases cell iron uptake to impact the release of pro-inflammatory mediators after particle exposure

**DOI:** 10.1038/s41598-023-30335-2

**Published:** 2023-03-09

**Authors:** Andrew J. Ghio, Joleen M. Soukup, Lisa A. Dailey, Victor L. Roggli

**Affiliations:** 1 Human Studies Facility, US Environmental Protection Agency, 104 Mason Farm Road, Chapel Hill, NC 27599-7315 USA; 2 Department of Pathology, Duke University Medical Center, Durham, NC USA

**Keywords:** Diseases, Respiratory tract diseases, Asthma, Chronic obstructive pulmonary disease, Respiratory distress syndrome

## Abstract

We tested the hypothesis that (1) mucus production can be included in the cell response to iron deficiency; (2) mucus binds iron and increases cell metal uptake; and subsequently (3) mucus impacts the inflammatory response to particle exposure. Using quantitative PCR, RNA for both MUC5B and MUC5AC in normal human bronchial epithelial (NHBE) cells decreased following exposures to ferric ammonium citrate (FAC). Incubation of mucus-containing material collected from the apical surface of NHBE cells grown at air–liquid interface (NHBE-MUC) and a commercially available mucin from porcine stomach (PORC-MUC) with iron demonstrated an in vitro capacity to bind metal. Inclusion of either NHBE-MUC or PORC-MUC in incubations of both BEAS-2B cells and THP1 cells increased iron uptake. Exposure to sugar acids (*N*-acetyl neuraminic acid, sodium alginate, sodium guluronate, and sodium hyaluronate) similarly increased cell iron uptake. Finally, increased metal transport associated with mucus was associated with a decreased release of interleukin-6 and -8, an anti-inflammatory effect, following silica exposure. We conclude that mucus production can be involved in the response to a functional iron deficiency following particle exposure and mucus can bind metal, increase cell uptake to subsequently diminish or reverse a functional iron deficiency and inflammatory response following particle exposure.

## Introduction

Following particle exposure, host iron previously employed in essential processes is sequestered at the particle surface with a functional cell deficiency of the metal resulting^1^. Both inorganic and carbonaceous particles demonstrate this capacity to complex and accumulate host cell iron. If enough iron is lost to the particle, cell death can result. Prior to death, exposed cells respond to the loss of its iron to complexation by surface functional groups of particles with attempts to acquire additional metal. This response can include an elevation in expression of metal importers and storage proteins (e.g. divalent metal transporter-1 (DMT1) and ferritin) to diminish or reverse the functional iron deficiency^1^. The initiation of biological effects by particles (e.g., inflammation) corresponds with these changes in cell iron homeostasis^1–3^.

Fundamental in the human response to particle exposure is an increased production and release of mucus in the respiratory tract^4^. The most common causes of chronic bronchitis, that is daily cough with sputum production (including mucus) for at least three months for 2 years in a row, are particle-related exposures including cigarette smoking and occupational particles (e.g., smoker’s bronchitis and industrial bronchitis respectively). Mucus accumulation in these patients can be associated with critical outcomes such as lung function, health-related quality of life, hospitalizations, mortality, exacerbations of chronic obstructive pulmonary disease (COPD), and pulmonary function decline in idiopathic pulmonary fibrosis^4^.

Mucus is comprised of water, ions, and a variety of macromolecules which possess protective functions. Among the structural and functional components of mucus, mucins are complex, high molecular weight glycoproteins which include a protein backbone with covalently O-linked oligosaccharides^5^. While the structures of mucins and their regulation are not fully recognized, it is accepted that polysaccharides account for greater than 70% of their mass and there is a high content of sugar acids including sialic acids (a family of derivatives of neuraminic acid) and uronates^6,7^. These polysaccharides contain both hydroxyl and carboxylate functional groups which create a negative charge at the interface with an epithelial cell membrane. Comparable to other hydroxycarboxylates (e.g., bacterial siderophores), the polysaccharides complex metals including iron.

We tested the hypothesis that (1) mucus production can be included in the cell response to iron deficiency; (2) mucus binds iron and increases cell metal uptake; and (3) mucus subsequently can impact the inflammatory response to a particle exposure.

## Results

### Quantitative PCR (qPCR) for MUC5B and 5C

**Figure 1:**
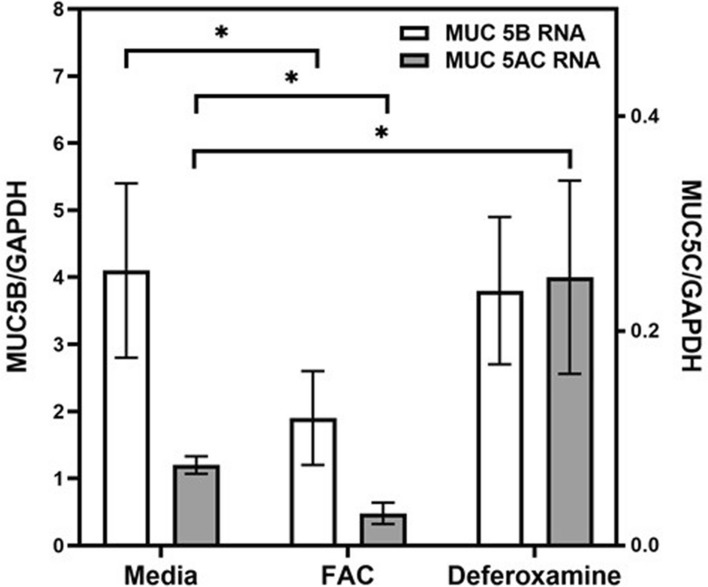
qPCR for MUC5B and MUC5C. RNA for MUC5B and MUC5AC was quantifiable in NHBE cells. After exposure to FAC, RNA for MUC5B and MUC5AC decreased while deferoxamine had the opposite effect for MUC5AC. *Increased or decreased relative to media (p < 0.05).

Respiratory tract gel-forming mucins (e.g., MUC5AC and MUC5B) can be associated with hypertrophy and hyperplasia of bronchial epithelium^8–10^. qPCR revealed measurable quantities of RNA for MUC5B and MUC5AC in normal human bronchial epithelial (NHBE) cells (Fig. 1).

RNA for MUC5B and MUC5AC decreased significantly after ferric ammonium citrate (FAC) (analyzed using separate T tests). Deferoxamine had the opposite effect for MUC5AC with elevations (T test). Results supported an association of MUC5B and MUC5AC expression by NHBE cells with iron availability.

### Characterization of mucus-containing materials

Two different mucus-containing materials were used in studies. The first was harvested from NHBE cells grown at air–liquid interface (NHBE-MUC) while the second was a commercially available porcine stomach mucin (PORC-MUC). Both had measurable quantities of protein (Table 1). There was ferritin in the NHBE-MUC but none in the PORC-MUC. Transferrin could not be determined in either mucus. NHBE-MUC had concentrations of metals which were increased relative to PORC-MUC reflecting the collection, and subsequent contamination, in BEGM. In addition, there were quantifiable concentrations of interleukin (IL)-1β, IL-6, IL-8, and tumor necrosis factor (TNF)-α in NHBE-MUC (1.5 ± 1.8, 5.6 ± 2.2, 20.9 ± 7.8, and 1.8 ± 1.3 pg/mL respectively). There was no measurable concentration of any mediator in the PORC-MUC.

**Table 1:** Composition of mucus from NHBE cultures and porcine mucus.

	NHBE-MUC	PORC-MUC
Total protein (µg/mL)	245 ± 51	634 ± 28
Ferritin (ng/mL)	31 ± 8	Below detectable level
Transferrin (µg/dL)	Below detectable level	Below detectable level
Calcium (ppm)	89.18 ± 8.28	1.56 ± 0.18
Magnesium (ppm)	25.66 ± 2.56	0.49 ± 0.03
Iron (ppm)	0.44 ± 0.03	0.31 ± 0.01
Zinc (ppm)	0.18 ± 0.03	0.08 ± 0.01
Copper (ppm)	Below detectable level	Below detectable level
Manganese (ppm)	Below detectable level	Below detectable level
Nickel (ppm)	Below detectable level	Below detectable level

### In vitro binding of mucus with iron

**Figure 2:**
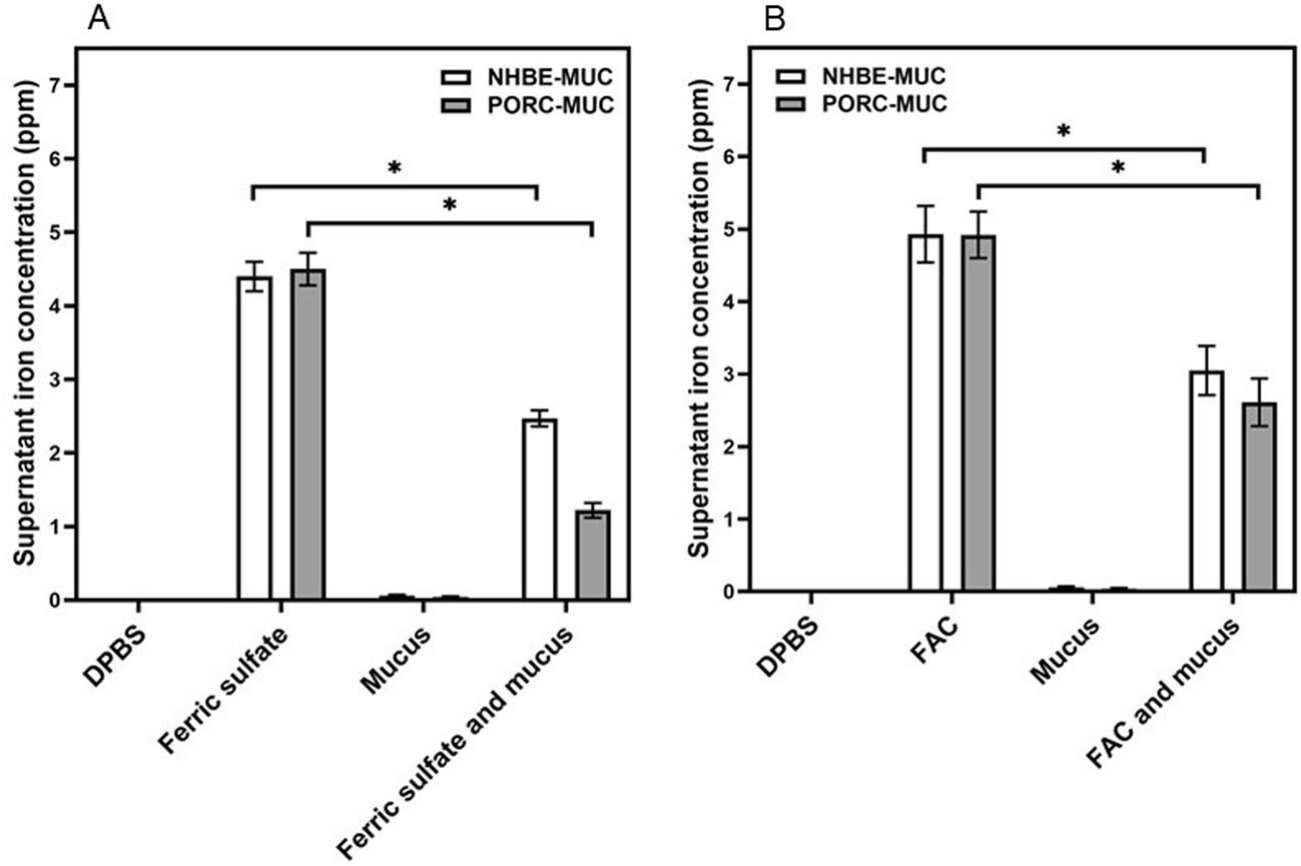
In vitro iron binding by NHBE-MUC and PORC-MUC. NHBE-MUC and PORC-MUC were exposed to either Fe_2_(SO_4_)_3_ (**A**) or FAC (**B**) for 15 min with agitation. Exposures demonstrated mucus significantly decreased iron concentrations with metal sequestration in the mucus pellet. Separate one way ANOVAs were employed for NHBE-MUC and PORC-MUC. *Decreased relative to ferric sulfate or FAC (p < 0.05).

Incubation of NHBE-MUC and PORC-MUC with ferric sulfate and FAC for 15 min demonstrated sequestration of iron by both (Fig. 2A,B respectively).

Greater concentrations of iron were bound by both NHBE-MUC and PORC-MUC after incubation of the mucus with ferric sulfate compared to FAC. Incubations confirmed an in vitro capacity of a mucus to sequester iron. Relative to NHBE-MUC, PORC-MUC had a significantly greater capacity for binding iron from ferric sulfate but not from FAC. These incubations confirmed an in vitro capacity of a mucus to bind iron.

### In vivo binding of mucus with iron

**Figure 3:**
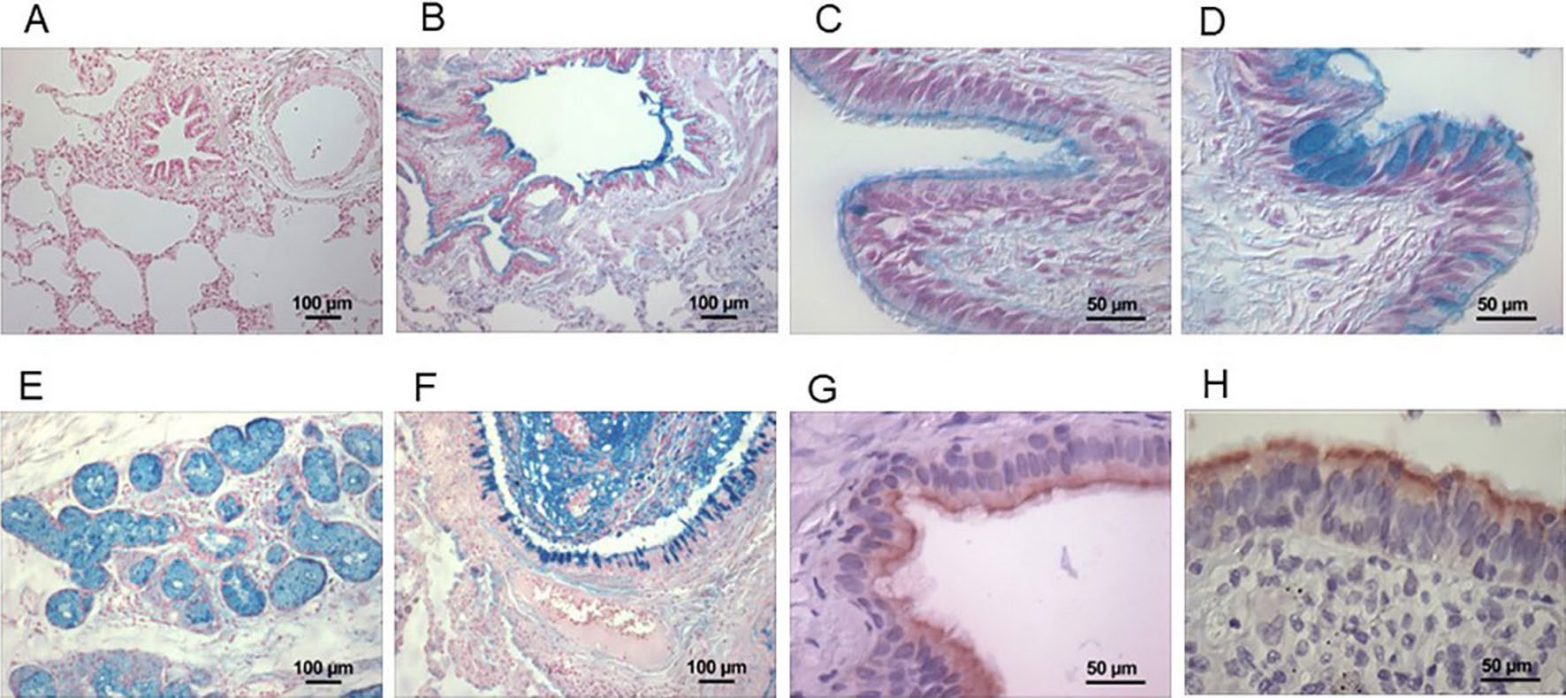
In situ iron binding by mucus and immunohistochemistry. Lung tissue from six patients diagnosed with COPD was stained for iron (Perls’ stain) and iron-binding (Hale’s stain). Photomicrographs were obtained using With Perls’ stain of the tissues, there was no airway iron associated with mucus (**A**). However, the Hale’s stain of every lung tissue demonstrated iron uptake (blue) at the apical surface of the airway epithelium (**B**,**C**), in goblet cells in the epithelium (**D**), and in the submucosal glands (**E**). Accumulation of mucus in inflamed, proximal airways of patients with COPD similarly corresponded to metal uptake with Hale’s stain (**F**). Immunohistochemical staining for DMT1 (**G**) and ferritin (**H**) demonstrated both (brown) at the apical membrane of the ciliated airway epithelium. These stains localized the expression of iron transport and storage proteins to the apical membrane, immediately adjacent to the mucus. Original magnification: (**A**,**B**,**E**) and (**F**) × 100; (**C**,**D**,**G**) and (**H**) × 400.

In patients with COPD, the Perls’ stain did not demonstrate iron in mucus of the lung (Fig. 3A). In these patients, the Hale’s stain demonstrated iron binding in the airway lining fluid at the apical surface of the airway epithelium (Figs. 3B,C), in the goblet cells of the airway epithelium (Fig. 3D), and in the submucosal glands (Fig. 3E) supporting a capacity of mucus for in situ binding of iron in the human lung. Accumulation of mucus in the inflamed, proximal airway of a patient with COPD similarly corresponded to metal uptake with Hale’s stain (Fig. 3F). In COPD patients, staining for the iron importer DMT1 (Fig. 3G) and storage protein ferritin (Fig. 3H) showed localization of both to the apical membrane of the airway epithelium immediately adjacent to the mucus.

This histology supported an in situ potential of mucus to bind iron at the apical membrane of respiratory epithelium which co-localized with proteins involved in both transport and storage of the protein.

### Mucus and iron uptake

**Figure 4:**
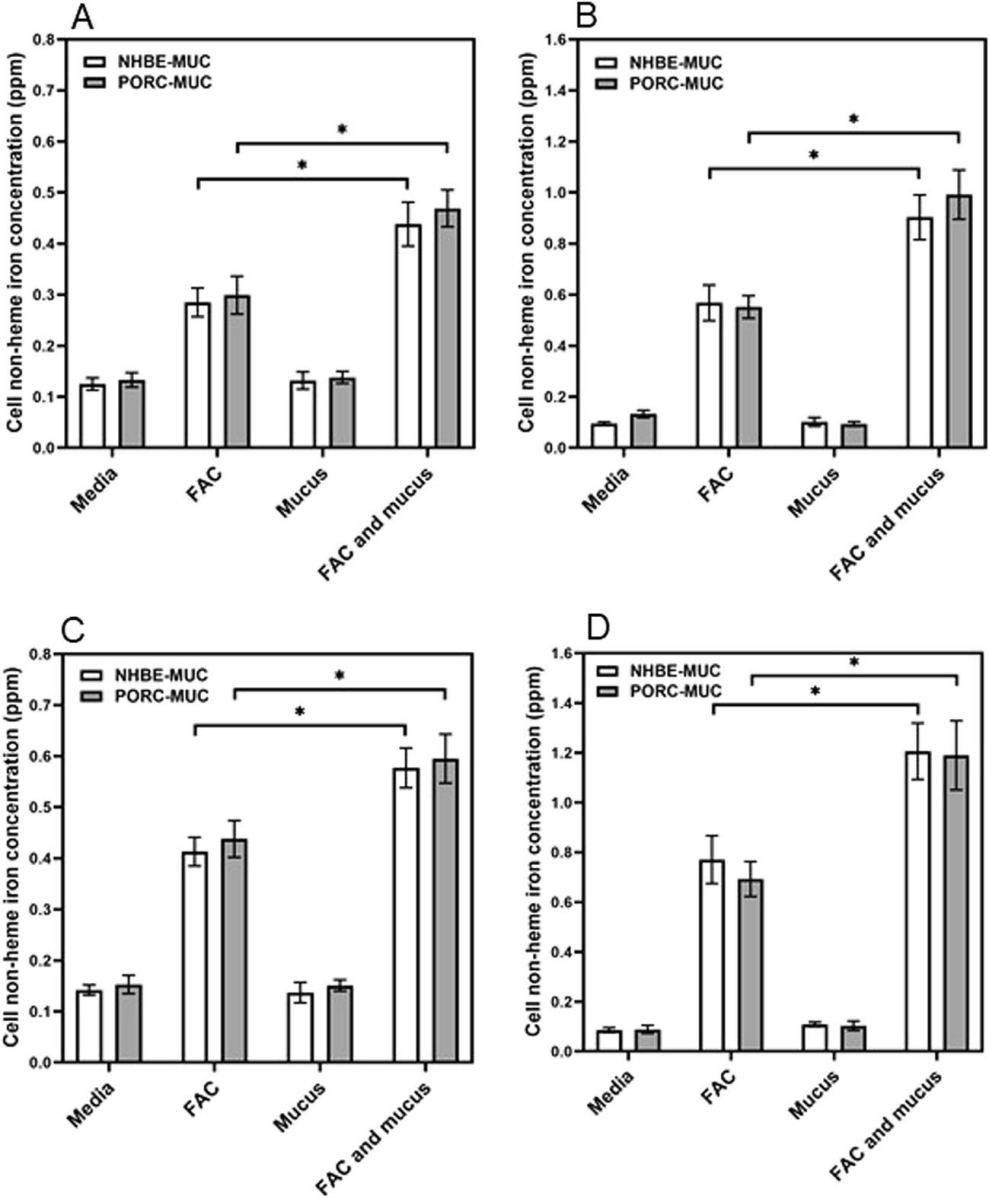
Cell iron concentrations after exposures to mucus and iron. With FAC exposure, cells imported iron. With inclusion of either NHBE-MUC or PORC-MUC, concentrations of iron in BEAS-2B cells significantly increased at both 4 and 24 h (**A**,**B** respectively) to levels greater than those observed with FAC alone. Similarly, THP1 cells imported significantly greater concentrations of FAC at 4 and 24 h (**C**,**D** respectively) with inclusion of either NHBE-MUC or PORC-MUC relative to metal alone. Separate one way ANOVAs were employed for NHBE-MUC and PORC-MUC. *Increased relative to FAC (p < 0.05).

**Figure 5:**
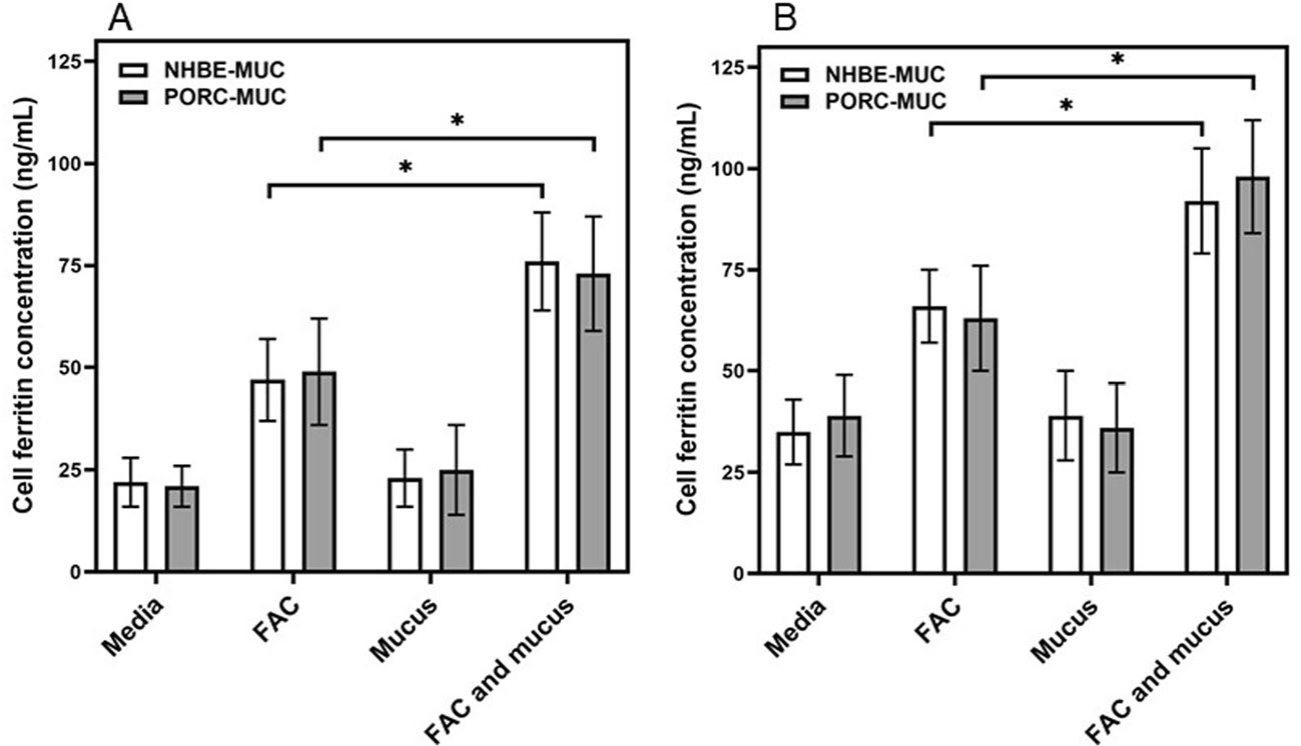
Cell ferritin concentrations after exposures to mucus and iron. With inclusion of either NHBE-MUC or PORC-MUC, cell ferritin concentrations were significantly increased in BEAS-2B cells (**A**) and THP-1 cells (**B**) relative to metal alone. Separate one way ANOVAs were employed for NHBE-MUC and PORC-MUC. *Increased relative to FAC (p < 0.05).

BEAS-2B cells, an immortalized line of normal human bronchial epithelium, exposed to FAC for both 4 and 24 h demonstrated elevated non-heme iron concentration (Fig. 4A,B). Inclusion of either NHBE-MUC or PORC-MUC with the metal further increased cell non-heme iron concentration (Fig. 5A,B). Incubation of THP1 cells, a monocyte-like cell line, with for 4 and 24 h increased non-heme iron concentration to levels greater than those for BEAS-2B cells (Fig. 4C,D). Inclusion of either NHBE-MUC or PORC-MUC also increased iron uptake to higher levels in THP1 cells (Fig. 4C,D).

Exposure of BEAS-2B cells to FAC for 24 h resulted in a cell ferritin concentration which approximately doubled (Fig. 5A). Addition of both NHBE-MUC and PORC-MUC significantly elevated cell ferritin further (Fig. 5A). Comparably, THP1 cells exposed to FAC for 24 h increased cell ferritin and inclusion of NHBE-MUC and PORC-MUC elevated these levels (Fig. 5B).

Exposure to mucus increased cell iron uptake. Elevations in ferritin after incubations with mucus confirmed that the increased metal concentration was intracellular.

### Sugar acids and iron uptake

**Figure 6:**
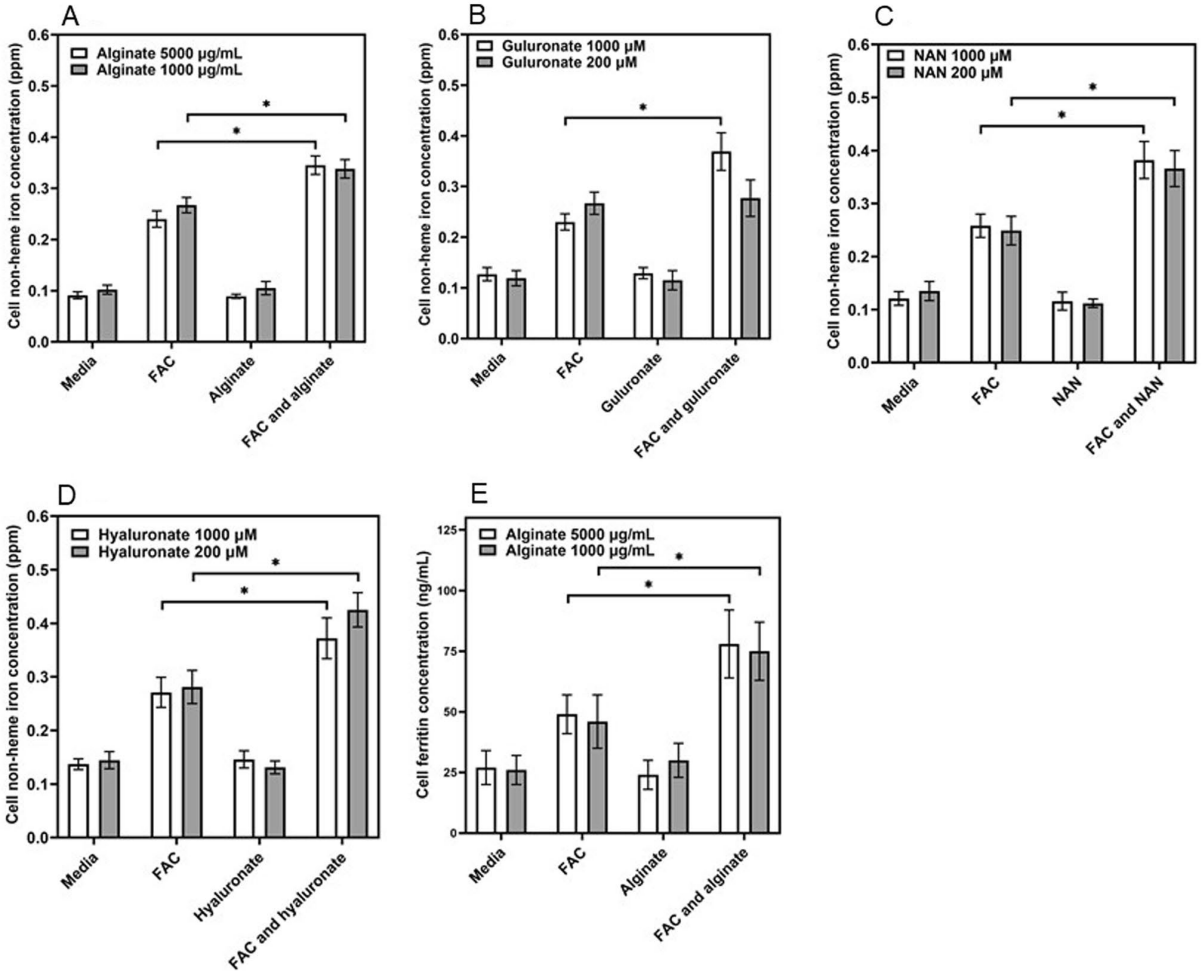
Cell iron and ferritin concentrations after exposures to NAN, alginate, guluronate, and hyaluronate. With inclusion of NAN, alginate, guluronate, and hyaluronate (**A**–**D** respectively), concentrations of iron in BEAS-2B cells significantly increased at 4 h levels greater than those observed with FAC alone. Relative to iron alone, cell ferritin in BEAS-2B cells was increased at 24 h with inclusion of alginate (**E**). One way ANOVA was employed. *Increased relative to FAC (p < 0.05).

BEAS-2B cells were exposed to N-acetyl neuraminic acid (NAN), alginate, guluronate, and hyaluronate without and with FAC. While FAC increased cell non-heme iron concentrations, inclusion of NAN, alginate, guluronate, and hyaluronate further elevated levels of cell metal (Fig. 6A–D). Cell incubations with alginate and FAC demonstrated increased ferritin concentrations at 24 h (Fig. 6E).

Cell exposure to sugar acids, comparable to components of mucus, increased iron uptake. Changes in ferritin with alginate confirmed that the iron was intracellular.

### Mucus and release of inflammatory mediators

**Figure 7:**
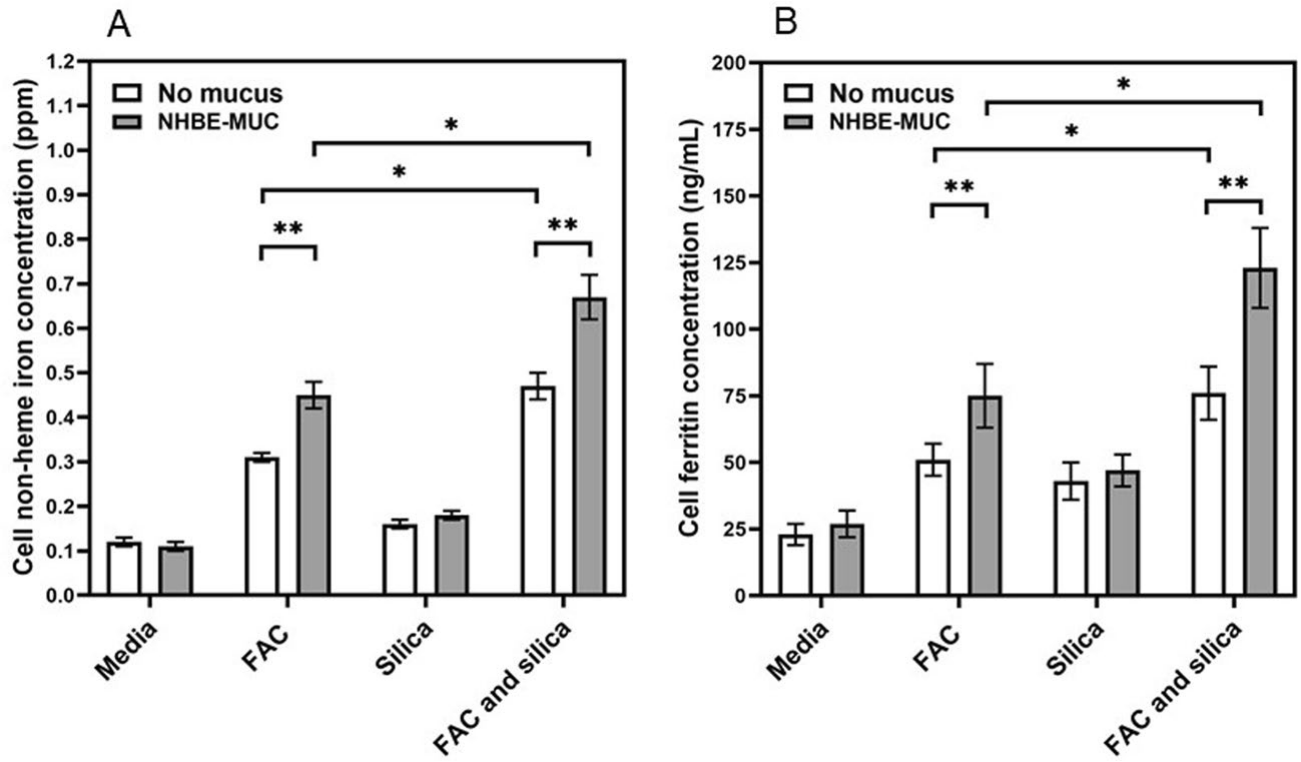
Cell iron and ferritin concentrations after exposures to mucus, iron, and silica. With FAC exposure, BEAS-2B cells imported iron (**A**). Those cells exposed to iron, mucus, and silica had the highest levels of iron. In a comparable manner, those exposures of BEAS-2B cells to iron, mucus, and silica demonstrated the greatest ferritin concentrations (**B**). One way ANOVA was employed. *Increased relative to FAC (p < 0.05); **increased relative to without mucus (p < 0.05).

Exposure of BEAS-2B cells to silica did not increase non-heme iron concentration while co-incubation with FAC did (Fig. 7A). However, exposure to silica with both NHBE-MUC and FAC further elevated cell non-heme iron levels (Fig. 7B).

**Figure 8:**
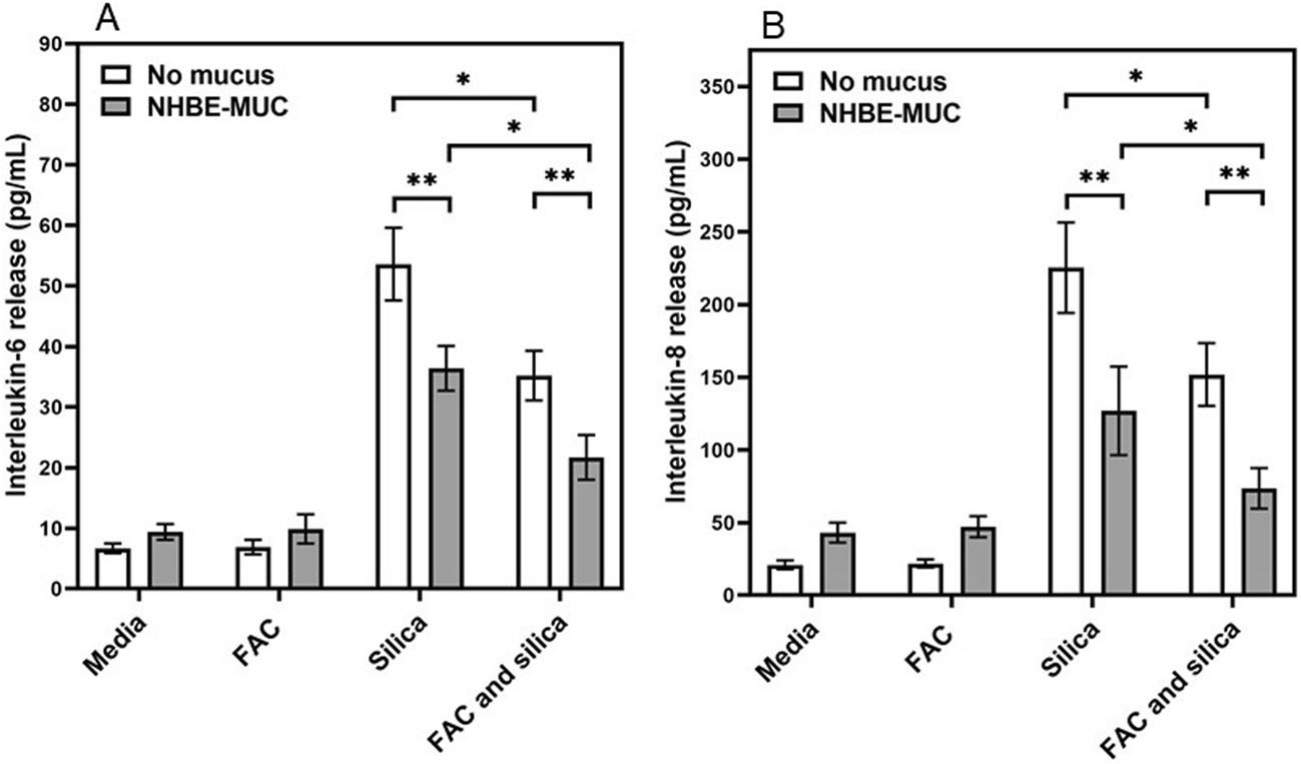
Release of inflammatory mediators after exposures to iron, mucus, and silica. While silica increased cell release of IL-6 (**A**) and IL-8 (**B**), iron and mucus both decreased these levels. One way ANOVA was employed. *Decreased relative to silica without iron (p < 0.05); **decreased relative to without mucus (p < 0.05).

BEAS-2B cell exposures to silica were repeated and cytokine release in the media quantified. The cell release of IL-1β and TNF-α were not impacted by cell incubation with silica. However, there were elevations in the inflammatory cytokines IL-6 and IL-8 following particle exposure (Fig. 8A,B respectively). The inclusion of FAC decreased levels of these mediators (Fig. 8A,B). Similarly, the inclusion of NHBE-MUC further decreased levels of IL-6 and IL-8 in the incubations of BEAS-2B with silica (Fig. 8A,B).

Increased metal transport with greater iron uptake into the BEAS-2B cells after incubation with mucus was associated with a decreased release of mediators (an anti-inflammatory effect).

## Discussion

In this investigation, the expression of mucins corresponded to iron availability. This relationship supports the postulate that mucin production can be involved in an attempt by the respiratory tract to reverse metal deficiency such as that following metal complexation and sequestration by inhaled particles (e.g., cigarette smoke particle and silica). This is comparable to the production of glycoproteins in the capsules of unicellular organisms when iron availability is varied^11–13^.

A capacity for mucus to bind iron both in vitro and in situ was confirmed. Polysaccharides are recognized to form coordination complexes with metal ions^14^. Such complexation of iron by sugar acids (e.g. sialic acid and polyuronates) has been shown to involve both carboxyl and hydroxyl groups^15^. The binding constant between a polyuronate (e.g. alginate) and iron(III) can approximate 10^4^/M^16^. The number of binding sites per molecule for alginate has been estimated to be 66 indicating that these polysaccharides can potentially bind a very large number of iron ions. Some component(s) of mucus has been demonstrated to have an ability to bind numerous metals including iron, calcium, lead, cadmium, aluminum, copper, technetium, and mercury^17,18^. The affinity of pig gastric mucus glycoprotein for cations varies but that for iron is greater relative to other metals^19^. A metal-binding activity by mucus has also been demonstrated using histologic methodology^20–22^. Colloidal iron stains, such as Hale’s stains, correlate this metal-binding to carboxylated mucopolysaccharides^23,24^. Capsules and biofilms potentially employ sugar acids as a cation sequestration mechanism. In these exchange processes, cations of higher charge (e.g. Fe^3+^) dominate the exchange sites on surfaces relative to monovalent cations in solution^25^. Comparable to capsules and biofilms, mucus bound iron likely due to components such as sugar acids.

Co-incubation of BEAS-2B cells with either mucus and iron increased metal concentrations in both BEAS-2B and THP-1 cells. These elevations in cell iron were reflected by equivalent increases in ferritin confirming that the metal had been transported intracellularly. This supports a role for mucus in promoting cell metal uptake. Exposure to sugar acids including NAN, alginate, guluronate, and hyaluronate displayed a comparable ability to increase cell metal concentrations. Sugar acids in capsular polysaccharides of unicellular organisms also facilitate iron acquisition^26^. Sugar acids and glycosaminoglycans, with almost all of the latter being polyuronates, demonstrate a similar potential for increasing metal delivery to human cells^27,28^. Comparable to mucus, polyuronate components at the soil–root interface participate in iron uptake and accumulation by plants^29^. In a living system, increased uptake of iron by mucus was demonstrated in animals given radiolabeled metal^30^. Some conflicting data exists with sugar acids actually decreasing cell iron uptake^31,32^. However, mucus also exhibits a capacity to increase cell and animal metal concentrations and this can be associated with specific components including sugar acids.

The ability of polysaccharides to complex metals can include bridges between the polymer chains leading to the formation of ionic cross-linked networks and structures which modifies the gel polymer networks. It is known that metal cations, including iron, trigger and accelerate gelation of polyuronates^33,34^. The mechanical properties of such a hydrogel can be changed after complexation with the metal cation^35,36^. With binding of available metal cations, there is crosslinking, and chain stiffening^37,38^. Metal cations, including iron, can also depolymerize polyuronates^39–43^. With iron deficiency, lyases are activated and these release oligomers from polysaccharides while the reverse is true with increased metal availability^44–46^. The synthesis and depolymerization of such polysaccharides provide polymeric units with very high number of binding sites and these can be utilized by living systems for metal uptake^47^. Accordingly, there are different mechanisms by which mucus could increase metal cell uptake. The reaction of the hydroxyl and carboxylate groups can increase the effective concentration of the metal immediately adjacent to the membrane and elevate iron uptake by the cells. Higher concentrations of calcium and iron precipitate polyuronates and therefore it is also possible that the complexation product of the polysaccharide with metal could fall out of solution resulting in cell endocytosis. Finally, cell enzymes might facilitate cleavage of the polymer into short oligosaccharides which, after binding to the membrane, would be followed by receptor-mediated uptake.

Particles utilize surface functional groups to sequester the metal from the cell thereby creating a metal deficiency. Mucus in the human lower respiratory tract of humans correlates with particle exposures and can provide a pathway to increase cell iron uptake to reverse the associated functional iron deficiency. Following exposures of BEAS-2B cells to silica, incubations with mucus did increase both cell iron concentrations and ferritin levels. Such modification in the reduction of available iron predicted that mucus would also diminish release of inflammatory mediators with particle exposure. Exposure of BEAS-2B cells to silica with co-incubations of mucus and iron was associated with decreased release of both IL-6 and IL-8. Such an inverse relationship between cell iron and endpoints of inflammation has been described. Changes in RNA and protein expression for pro-inflammatory mediators after particle can be diminished by cell pre-treatment with iron^2,48^. Following exposure to compounds and substances which impact metal homeostasis to elevate available iron, cells can show a diminished release of pro-inflammatory mediator^49,50^. The association between iron availability and inflammation supports mucus production being included in a host response to diminish or reverse functional iron deficiency after particle exposure and subsequently serving an anti-inflammatory role.

Unicellular organisms evolved efficient iron acquisition systems allowing survival in a deficient environment. In these microbials, metal biosorption occurs through a complexation of metal cations by polysaccharides using carboxyl, hydroxyl, carbonyl, and sulfate functional groups^51^. Bacterial cells surrounded by capsular polysaccharides have larger numbers of metal ion‐binding sites relative to non‐capsulated strains; these polysaccharides have abundant uronic acid subunits that can efficiently bind metal ions using their carboxyl groups with iron being preferred^26,52–54^. Polysaccharides function as organic ligands to bind and enhance iron bioavailability and may also act as a reservoir of the metal^26,55,56^. In response to environmental conditions, bacteria also synthesize biofilms which are multicellular, surface-associated microbial communities encased in an extracellular matrix which includes anionic polysaccharides with cation binding activity^57^. This can include alginate, a polyuronate. The development of such a biofilm is advantageous to survival and growth of the unicellular organism^58^. The anionic character of the polysaccharide gives biofilms cation exchange properties allowing the concentration and utilization of metals. As a result of this association, iron acquisition systems are expressed among biofilms^59^.

There are several limitations of this investigation. There is a lack of standardized materials available (i.e., mucus and mucins). While the PORC-MUC is convenient to use, physiological relevance has been questioned. The two mucus models employed in these studies are spatially heterogeneous (i.e., the components within the gel are not necessarily uniformly distributed) and they are difficult to use. Neither the NHBE-MUC nor the PORC-MUC is strictly representative of airway mucus which can include numerous other iron-related compounds (e.g. transferrin, lactoferrin, ferritin, and neutrophil gelatinase-associated lipocalin). In addition, it is recognized that the metal chelator employed in these studies (i.e. deferoxamine) is not specific for iron. Finally, Hale’s stain was used to distinguish mucus. This is a colloidal stain with the metal being bound by acid mucins. Other carboxylated and sulfated mucosubstances can possibly provide a positive Hale’s stain.

## Conclusions

We conclude that (1) mucus production can be included in the response to the functional iron deficiency following particle exposure and (2) mucus can bind metal, increase cell uptake, and subsequently diminish or reverse a functional iron deficiency and inflammatory response following particle exposure. It is proposed that increased mucus production in chronic bronchitis reverses a functional iron deficiency following the complexation and sequestration of this metal by particles and microbes. A comparable model for transport has previously suggested that mucin in the gastrointestinal tract serves to chelate metal for uptake by the intestinal cell^60^.

## Materials and methods

### Materials

There were two sources of mucus employed in these studies. The first was from the apical surface of NHBE cells grown at air–liquid interface (ALI)^61^. The methods for acquisition of NHBE cells were carried out in accordance with relevant guidelines and regulations and the protocol and informed consent were approved by the University of North Carolina School of Medicine Committee on Protection of the Rights of Human Subjects and by the U.S. Environmental Protection Agency. NHBE cells were obtained from healthy subjects (18–40 years of age) through bronchoscopy with bronchial brushings. These were maintained at air–liquid interface for more than 21 days allowing their differentiation into ciliated, mucus-producing cells (which occurred at approximately day 10 and later)^62^. After NHBE cells started to produce mucus, this material was aspirated from the apical chamber employing a Pasteur pipet, without addition of any fluid, during the 48 h between feedings. The harvested mucus (NHBE-MUC) was pooled and stored at − 80 °C. The second source of mucus employed in studies was commercially available mucin from porcine stomach (PORC-MUC) (catalog number M1778, Sigma; CAS number 84082-64-4)^63,64^. This was characterized as a partially purified powder with a molecular mass of 80–84% carbohydrate, 16–20% protein, and 0.5–1.5% bound sialic acid. PORC-MUC was stored at 2–8 °C as recommended and reconstituted in Dulbecco's Phosphate Buffered Saline (DPBS).

NHBE-MUC and PORC-MUC were characterized. Protein was determined using Bio-Rad 5× Protein Dye Reagent Concentrate (Benicia, CA). Ferritin and transferrin were measured using an immunoturbidimetric assay (Kamiya Biomedical Company, Seattle, WA) and an immunoprecipitin analysis (INCSTAR Corporation, Stillwater, MN) respectively. After digestion in 3 N HCl/10% trichloroacetic acid and digested at 70° C, calcium, magnesium, iron, zinc, copper, manganese, and nickel concentrations were measured using inductively coupled plasma optical emission spectroscopy (ICPOES; Model Optima 4300D; Perkin Elmer, Norwalk, CT) operated at wavelengths of 393.366, 280.271, 238.204, 206.200, 327.393, 257.610, and 231.604 nm respectively. Concentrations of IL-1β, IL-6, IL-8, and TNF-α were determined using immunoassays (MesoScale Discovery, Rockville, MD).

The silica used in studies was Min-U-Sil (5 micron diameter from U.S. Silica, Berkeley Springs, West Virginia). All other reagents were from Sigma (St. Louis, MO) unless specified otherwise.

### qPCR for MUC5B and MUC5AC

NHBE cells were cultured in 12 well plates in BEGM with supplements and exposed for 24 h to media alone, 200 μM FAC, and 50 μM deferoxamine. Total RNA was isolated using a Qiagen kit (Qiagen, Valencia, CA) and reverse transcribed to generate cDNA using a High Capacity cDNA Reverse Transcription kit (Applied Biosystems, Foster City, CA). Oligonucleotide primer pairs and fluorescent probes for MUC5B, MUC5AC, and GAPDH were designed using a primer design program (Primer Express; Applied Biosystems) and obtained from Integrated DNA Technologies (Coralville, IA). Quantitative fluorogenic amplification of cDNA was performed using the ABI Prism 7500 Sequence Detection System (Applied Biosystems), primer/probe sets of interest, and TaqMan Universal PCR Master Mix (Applied Biosystems). The relative abundance of GAPDH mRNA was used to normalize mRNA levels.

### In vitro binding of iron by mucus

To quantify the binding of iron by NHBE-MUC and PORC-MUC, exposures included (1) DPBS, (2) 50 μM Fe_2_(SO_4_)_3_ in DPBS, (3) either 100 μL NHBE-MUC or 1000 μg/mL PORC-MUC in DPBS, and (4) both 50 μM Fe_2_(SO_4_)_3_ and 100 μL NHBE-MUC or 1000 μg/mL PORC-MUC in DPBS. After 15 min incubation at 37 °C with agitation, the microfuge tubes were centrifuged (18,000*g*). Iron in the supernatant was measured using ICPOES operated at a wavelength of 238.204 nm. The experiment was repeated using 100 µM FAC as the source of metal rather than Fe_2_(SO_4_)_3._

### Tissue procurement, iron and colloidal iron stains, and immunohistochemistry

The study was reviewed and approved by the Duke University Institutional Review Board (Durham, NC). An existing cohort of patients diagnosed to have chronic obstructive pulmonary disease (COPD; some combination of chronic bronchitis and emphysema) was searched and six individuals identified. Blocks of lung tissue collected at autopsy were retrieved from archives.

Perls’ Prussian blue was employed to stain iron. Hale’s stain was used as an assay for in situ iron binding capacity^20^. The background stain was nuclear fast red. Tissue was stained for an iron importer and storage protein. Five micron tissue sections were cut, floated on a protein-free water bath, mounted on silane treated slides, and air-dried overnight. Sections were then deparaffinized and hydrated to 95% alcohol (xylene for 10 min, absolute alcohol for 5 min, and 95% alcohol for 5 min). Endogenous peroxidase activity was blocked with 0.6% H_2_O_2_ in absolute methanol for eight minutes. Slides were rinsed in 95% alcohol for 2 min, placed in deionized H_2_O, and washed in PBS. After treatment with Cyto Q Background Buster (Innovex Biosciences, Richmond, CA) for 10 min, slides were incubated with the primary antibody diluted in 1% bovine serum albumin for 45 min at 37 °C in PBS. Primary antibodies used in this investigation were to divalent metal transport 1 (DMT1) (generously provided by Dr. Funmei Yang of the University of Texas, San Antonio, TX) used at a dilution of 1:200 and ferritin (Dako, Carpinteria, CA) used at a dilution of 1:200. Slides were incubated with biotinylated linking antibody from Stat-Q Staining System (Innovex Biosciences) for ten minutes at room temperature, washed with PBS, and peroxidase enzyme label from Stat-Q Staining System (Innovex Biosciences) applied. After incubation for ten minutes at room temperature and washes with PBS, tissue sections were developed with 3,3′diaminobenzidine-tetrahydrochloride for three minutes at room temperature. Sections were counterstained with hematoxylin, dehydrated through alcohols, cleared in xylene and coverslipped using a permanent mounting media. Photomicrographs were obtained using a Nikon Eclipse E600 microscope (Tokyo, Japan) with 10×/40× objective lens coupled with QCapture software (QImaging, Surrey, British Columbia, Canada).

### Cell cultures

BEAS-2B cells were employed in in vitro studies of iron uptake, ferritin levels, and release of inflammatory mediators. This is an immortalized line of normal human bronchial epithelium derived by transfection of primary cells with SV40 early-region genes. BEAS-2B cells were grown to 90–100% confluence on uncoated plastic 12-well plates in keratinocyte growth medium (KGM; Clonetics) which is essentially MCDB 153 medium supplemented with 5 ng/mL human epidermal growth factor, 5 mg/mL insulin, 0.5 mg/mL hydrocortisone, 0.15 mM calcium, bovine pituitary extract, 0.1 mM ethanolamine and 0.1 mM phosphoethanolamine. Fresh medium was provided every 48 h.

THP1 cells, a monocyte-like cell line, were also used in in vitro investigation. These were cultured in 75-cm^2^ tissue culture flasks using RPMI-1640 (Invitrogen, Carlsbad, CA, USA) supplemented with 10% serum (Invitrogen, Carlsbad, CA, USA) and gentamicin solution (20 μg/mL; Sigma, St. Louis, MO, USA). Incubations were in RPMI-1640 supplemented with serum and gentamicin.

### Cell iron uptake after exposures to iron and mucus

BEAS-2B cells were grown in 12-well plates and exposed (3 wells per plate each) to (a) media alone, (b) 200 μM FAC, (c) either 100 μL NHBE-MUC or 1000 μg/mL PORC-MUC, and (d) both 200 μM FAC and 100 μL NHBE-MUC or 1000 μg/mL PORC-MUC. After 4 and 24 h incubation, the cells were gently washed, scraped into 1.0 mL of 3 N HCl/10% trichloroacetic acid and digested at 70 °C. Non-heme iron concentrations were determined using ICPOES.

Experiments were repeated using THP-1 cells (1.0 × 10^6^/mL) exposed to (a) media alone, (b) 200 μM FAC, (c) either 100 μL NHBE-MUC or 1000 μg/mL PORC-MUC, and (d) both 200 μM FAC and 100 μL NHBE-MUC or 1000 μg/mL PORC-MUC. After 4 and 24 h incubation, the cells were washed, collected into 10% trichloroacetic acid dissolved in 1.0 mL of 3 N HCl, and digested at 70 °C. Non-heme iron concentrations were determined using ICPOES.

### Cell ferritin concentrations

To examine the effect of NHBE-MUC and PORC-MUC on iron uptake, BEAS-2B cells were grown in 12-well plates and exposed (3 wells per plate each) to (a) media alone, (b) 200 μM FAC, (c) either 100 μL NHBE-MUC or 1000 μg/mL PORC-MUC, and (d) both 200 μM FAC and either 100 μL NHBE-MUC or 1000 μg/mL PORC-MUC. After 24 h incubation, the media was removed, cells were scraped into 0.5 mL PBS, and disrupted using five passes through a small gauge needle. The ferritin concentrations in the lysates were quantified using an immunoturbidimetric assay (Kamiya Biomedical Company).

THP1 cells (1.0 × 10^6^/mL) were exposed to (a) media alone, (b) 200 μM FAC, (c) either 100 μL NHBE-MUC or 1000 μg/mL PORC-MUC, and (d) both 200 μM FAC and 100 μL NHBE-MUC or 1000 μg/mL PORC-MUC. After 24 h incubation, the cells were washed, collected into 0.5 mL PBS, disrupted, and ferritin measured (Kamiya Biomedical Company).

### Cell iron uptake and ferritin after exposures to iron and *N*-acetyl neuraminic acid, sodium alginate, sodium guluronate, and sodium hyaluronate

BEAS-2B cells were grown in 12-well plates and exposed (3 wells per plate each) to (a) media alone, (b) 200 μM FAC, (c) 1000 μg/mL NAN (the predominant sialic acid in human cells and respiratory secretions), 1000 μg/mL sodium alginate (a polymer composed of mannuronate and guluronate monosaccharides), 1000 μM sodium guluronate (a uronate), or 1000 μM sodium hyaluronate (a polymer of disaccharides composed of glucuronate and *N*-acetyl-d-glucosamine) and (d) both 200 μM FAC and 1000 μg/mL NAN, 1000 μg/mL sodium alginate, 1000 μM sodium guluronate, or 1000 μM sodium hyaluronate. After 24 h incubation, the cells were gently washed, scraped into 10% trichloroacetic acid dissolved in 1.0 mL of 3 N HCl, digested at 70 °C, and non-heme iron concentrations were determined using ICPOES operated at a wavelength of 238.204 nm. Exposures of the BEAS-2B cells were repeated to (a) media alone, (b) 200 μM FAC, (c) 1000 μg/mL sodium alginate, and (d) both 200 μM FAC and sodium alginate for 24 h, the media was removed, cells were scraped into 0.5 mL DPBS and disrupted, and the ferritin concentrations quantified using an immunoturbidimetric assay.

### Iron uptake and inflammatory response after particle exposure

To examine the effect of NHBE-MUC on the inflammatory response to particle, BEAS-2B cells were grown in two 12-well plates and exposed (3 wells per plate each) to (a) media alone, (b) 200 μM FAC, (c) 100 μL NHBE-MUC, and (d) both 200 μM FAC and 100 μL NHBE-MUC. After 24 h, all wells in one plate were treated with 100 μL media while all wells in the other plate were treated with 100 μg silica in 100 μL media. After 4 h incubation, the cells were gently washed, scraped into 10% trichloroacetic acid dissolved in 1.0 mL of 3 N HCl and digested at 70 °C. Non-heme iron concentrations were determined using ICPOES.

The exposures of the BEAS-2B cells were repeated. After 24 h, aliquots of supernatant were obtained for determination of inflammatory cytokines. Concentrations of IL-1β, IL-6, IL-8, and TNF-α in cell media were measured using immunoassays.

### Statistics

Data are expressed as mean values ± standard deviation unless specified otherwise. The minimum number of replicates for all measurements was six. Differences between two and multiple groups were compared using T-tests of independent means and one-way analysis of variance (ANOVA) respectively. The post-hoc test employed was Tukey’s test. Two-tailed tests of significance were employed. Significance was defined at p < 0.05.

### Ethics approval

The protocol and informed consent for the acquisition of NHBE cells was approved by the University of North Carolina School of Medicine Committee on Protection of the Rights of Human Subjects and by the U.S. Environmental Protection Agency.

## Data Availability

All data generated or analyzed during this study are included in this manuscript.
